# SARS-CoV-2 Vaccination in Patients with Cancer and COVID-19 in Mexico

**DOI:** 10.3390/vaccines12101163

**Published:** 2024-10-12

**Authors:** Corazón Barrientos-Flores, Diana Vilar-Compte, Nancy Martínez-Rivera, Rodrigo Villaseñor-Echavarri, Alexandra Martin-Onraet

**Affiliations:** Department of Infectious Diseases, Instituto Nacional de Cancerología, Avenida San Fernando 22, Col. Sección 16 Belisario Domínguez, Tlalpan, Mexico City 14080, Mexico; coviru.cdjbf@gmail.com (C.B.-F.); dvilar@facmed.unam.mx (D.V.-C.); nancy.martinezr@alumno.buap.mx (N.M.-R.); rodrigovillaecha@gmail.com (R.V.-E.)

**Keywords:** COVID-19 vaccines, cancer, hematologic malignancies, solid tumors

## Abstract

Objectives: Vaccination is the best preventive measure for SARS-CoV-2 infection; however, efficacy is lower in cancer patients. During the pandemic period, Mexico was characterized by the use of seven different COVID-19 vaccine platforms, and oncologic patients were not prioritized for vaccination. We report the outcomes of COVID-19 in cancer patients after the beginning of the national vaccine campaign in Mexico. Methods: All patients with cancer and COVID-19 diagnosed at Instituto Nacional de Cancerología from 14 February 2021 to 28 February 2022 were included. Primary outcomes were the proportion of individuals who required hospital admission and/or invasive mechanical ventilation, according to the vaccination status; 30-day mortality; the period of infection; and other cancer-related variables. Results: A total of 691 patients were included; 524 (76%) had solid tumors (STs), and 167 (24%) had hematologic malignancies (HMs). Patients infected in the first two periods, had lower rates of vaccination and higher rates of mortality and hospitalization compared to those infected in the Omicron period. In the multivariate analysis, vaccination status was independently associated with hospitalization in patients with STs (aOR 0.38, 95%CI 0.19–0.75, *p* = 0.005), but it was not associated with invasive mechanical ventilation and 30-day mortality. In those with HMs, vaccination status was not associated with any outcome; in this group, only recent chemotherapy and time of infection were associated with invasive ventilation. Conclusions: Vaccination significantly reduced hospital admissions in patients with STs. Infections occurring during the Omicron period were associated with improved outcomes in both ST and HM patients. Despite having a lesser impact in immunosuppressed patients, vaccination is an essential strategy, and access to vaccination campaigns in patients with cancer needs to be prioritized.

## 1. Introduction

Patients with cancer and COVID-19 belong to one of the groups with the greatest risk for severe disease and death [1]. They have more than twice the risk of poor outcomes compared to the general population [2]. At the end of 2020, the first vaccines targeting SARS-CoV-2 were approved, and trials reported high safety, tolerability, and efficacy, with a reduction in mortality [3,4,5]. However, limited data were available among patients with active malignancies because of their ineligibility in most trials [6]. Only two vaccine trials enrolled patients with cancer, but these patients represented less than 5% of the entire sample [7]. Soon after the acceptance of mRNA SARS-CoV-2 vaccines, most international infectious and oncologic associations recommended vaccination of these patients [8,9,10]. Reports derived from the National COVID Cohort Collaborative (N3C) included 6860 cases, of which 1460 (21.3%) patients with cancer had significantly higher risks for breakthrough infection and severe outcomes compared with non-cancer patients, adjusting for age, sex, vaccine type, and vaccination date [11]. Compared with those with solid tumors (STs), individuals with hematological malignancies (HMs) were at an increased risk for breakthrough infections. Breakthrough risk was reduced after the second dose of the vaccine for all cancers [12].

Most studies evaluating vaccine efficacy in cancer patients only included mRNA vaccines and Ad26.COV2 [13]. There is less information on other vaccines used outside of Europe, Canada, and the US, such as the Cansino (AD5-nCOV), Sputnik, or Sinovac vaccines [14], all of which were used as part of the vaccination strategy in Mexico.

By the end of 2020, the first COVID-19 vaccines arrived in Mexico. Based on the Mexican government’s immunization strategy, healthcare workers were vaccinated first, followed by people ≥ 60 years. Cancer patients were not prioritized for early vaccination, and nor were they prioritized to receive a specific type of vaccine. Seven different COVID-19 vaccines were used, including mRNA vaccines, vector-based vaccines, and whole inactivated virus vaccines, such as the Sinovac vaccine [15].

The aim of this study was to describe the characteristics of COVID-19 infections in patients with cancer at the largest oncological institution in Mexico after the national rollout of COVID-19 vaccines.

## 2. Methods

### 2.1. Setting

Instituto Nacional de Cancerología (INCan) is a 133-bed tertiary care public cancer hospital for adult patients in Mexico City. From the beginning of the COVID-19 pandemic, the institution became a hybrid center, with an exclusive area for respiratory triage and COVID testing for patients with respiratory symptoms; a ward for COVID-19-related admissions, with 16 isolated beds; and an 8-bed critical care unit for those with severe COVID-19. In Mexico, vaccination against COVID-19 began on 24 December 2020, making healthcare workers the first group to receive the vaccine. In February 2021, COVID-19 vaccination was started for all ≥60 years and progressed thereafter by age group.

### 2.2. Study Design and Procedures

We conducted a retrospective study including all patients with cancer and a COVID-19 diagnosis between 14 February 2021, and 28 February 2022. Eligible cases were all adults > 18 years old with current or prior history of cancer and laboratory-confirmed SARS-CoV-2 infection, either confirmed by polymerase chain reaction (PCR) or a lateral flow antigenic test. We also included asymptomatic patients diagnosed through screening protocols. At that time at INCan, all patients undergoing surgery or receiving chemotherapy were screened for SARS-CoV-2 with a PCR. All data were collected from electronic medical charts and from prospectively obtained information from local COVID-19 databases. Demographic, oncological variables, and cancer-related treatment were included. Data on COVID-19 presentation, vaccination status, treatment received, and outcomes were assessed (data are available in a Supplementary Excel File).

### 2.3. Study Definitions

Patients were categorized as fully vaccinated at the time of SARS-CoV-2 infection when two or three doses of vaccine had been administered before a diagnosis of COVID-19. Unvaccinated patients were defined as those who had not received or had just received one dose of a COVID-19 vaccine at the time of the infection. This classification was established based on evidence of incomplete protection and lower efficacy in patients with cancer not fully vaccinated [13]. Patients vaccinated with one dose of Cansino were classified as not fully vaccinated considering the information that was published on the need for a booster for the vaccine [16].

The type of neoplasia was categorized as ST or HM. Active cancer was defined as patients with newly diagnosed cancer and patients with ongoing treatment. Patients were classified as having advanced cancer if metastatic disease or cancer recurrence were documented at SARS-CoV-2 diagnosis. Lymphomas were staged according to Lugano criteria [17].

For the purpose of the study, anticancer therapy was classified as cytotoxic chemotherapy and immunotherapy.

Severe COVID-19 was defined by SpO_2_ < 90% on room air, a ratio of arterial partial pressure of oxygen to fraction of inspired oxygen (PaO_2_/FiO_2_) < 300 mm Hg, a respiratory rate > 30 breaths/min, or lung infiltrates > 50% [8,18].

For study purposes, we classified the patients according to the following three COVID-19 waves: the first one took place from 1 February to 31 May 2021, the second one took place from 1 June to 31 November 2021 (corresponding to the Delta Wave), and the third one took place from 1 December 2021 to 28 February 2022, corresponding to Omicron. This classification was carried out according to the national reports of the main changes in variants in Mexico [19].

### 2.4. Outcomes

The main outcomes of the study were the proportion of patients who required hospital admission, critical care, and/or invasive mechanical ventilation (IMV), and 30-day mortality.

### 2.5. Statistical Analysis

Descriptive data are presented as frequency and proportions. For continuous variables, we use mean and standard deviation or median and interquartile range (IQR) as appropriate.

A univariate analysis was carried out to compare the clinical characteristics according to vaccination status and the type of cancer. Patients with unknown vaccination were excluded from the analysis. Student’s T test or the Mann–Whitney U test was used for quantitative variables. The chi-square test or Fisher’s exact test was used to compare qualitative variables. Odds ratios (ORs) and 95% confidence intervals (95%CIs) were calculated. The outcomes of interest were compared according to vaccination status and type of neoplasia. To account for potential confounders, we performed a multivariate analysis including age, recent chemotherapy, comorbidities, and the timing of infection, excluding asymptomatic patients. Data analysis was carried out with the statistical package STATA 16.0.

## 3. Results

We included 691 patients with cancer and COVID-19: 524 (76%) with STs, and 167 (24%) with HMs. Breast cancer was the most common solid tumor (35%), followed by gynecological (19%) and gastrointestinal (15%) tumors. Non-Hodgkin lymphoma was the most frequent hematologic malignancy (43%), followed by acute leukemias (25%) and plasma cell neoplasia (14%) (Figure S1).

Table 1 shows the demographic and baseline clinical characteristics of patients with cancer and COVID-19 overall and by tumor group. Patients with HMs were younger than patients with STs (49 yo, IQR 35–62 vs. 52 yo, IQR 43–63; *p* = 0.01), and there were more patients on recent chemotherapy in the HM group (50% vs. 35%, *p* < 0.001). Regarding comorbidities, obesity was more frequent in the ST group (23% vs. 14%, *p* = 0.01), whereas HIV was more frequent in the HM group (8% vs. 2%, *p* < 0.001), as was chronic renal failure (3% vs. 0.3%, *p* = 0.003).

Advanced-stage cases, defined as stage III and IV according to the Lugano classification [18], were reported in 89% of patients with lymphoma. Eighteen patients (3%) had a history of hematopoietic stem cell transplantation (HSCT) and 27 (3.9%) received rituximab within the last month before COVID-19 diagnosis, with a median of 26 days (IQR 11–114).

Overall, 265 patients (38%) received cytotoxic chemotherapy in the month prior to COVID-19 diagnosis; 113 patients (16.3%) were classified as cancer-free, and 54 (8%) were on palliative care.

### 3.1. Vaccination Status

One hundred eighty-six patients (27%) had not received a single dose of a COVID-19 vaccine. For 70 (10%) patients, we were unable to confirm their vaccination status. Overall, 104 patients (17%) received one dose, 261 (42%) received two doses, and 70 (11%) had three doses at COVID-19 diagnosis. For 437 patients (63.2%), information on the type of vaccine was available. Most patients (60%) received a viral vector COVID-19 vaccine. Overall, 40% of patients were vaccinated with AZD, 23% with BNT162B2, and 18% with Sputnik-V. Other vaccines included the Sinovac (10%), AD5-nCOV (6%), mRNA-1273 (1%), and JNJ-78436735 (1%) vaccines.

Fully vaccinated patients were eight years older than the non-vaccinated ones (55 yo, IQR 47–66 vs. 47 yo, IQR 37–58) (*p* < 0.01) and had a higher proportion of comorbidities such as Diabetes and hypertension (18% vs. 11%, *p* = 0.012, and 28% vs. 14%, *p* < 0.001, respectively). Non-vaccinated patients were more frequently under active cytotoxic chemotherapy (61% vs. 49%, *p* < 0.001) compared to the vaccinated group (Table S1).

### 3.2. COVID-19 Characteristics

Overall, the median time of symptoms to COVID-19 diagnosis was 3 days (range 2–6). Mild COVID-19 was found in 414 (60%) patients. Overall, 138 (20%) patients presented with severe or critical disease; asymptomatic cases were diagnosed in 65 (9.4%) patients, of which 58 were identified by preoperative screening. Vaccinated patients had less severe COVID-19 compared with unvaccinated patients. The patients with HMs had a more severe clinical presentation of COVID-19 diagnosis compared to the patients with STs: 15% of patients with STs had asymptomatic disease, compared with 2% of patients with HMs (*p* < 0.01), and 13% of ST group had severe COVID-19, compared to 32% in the HM group (*p* < 0.01). The distribution of COVID-19 severity by type of neoplasia and vaccination status is shown in Figure S2. Regarding treatment, 170 (25%) patients were prescribed dexamethasone, 210 (30%) used inhaled steroids, 8 (1%) were prescribed baricitinib, 29 (4%) were prescribed remdesivir, 7 (1%) were prescribed convalescent plasma, and 2 (0.2%) were prescribed tocilizumab.

**Figure 1:**
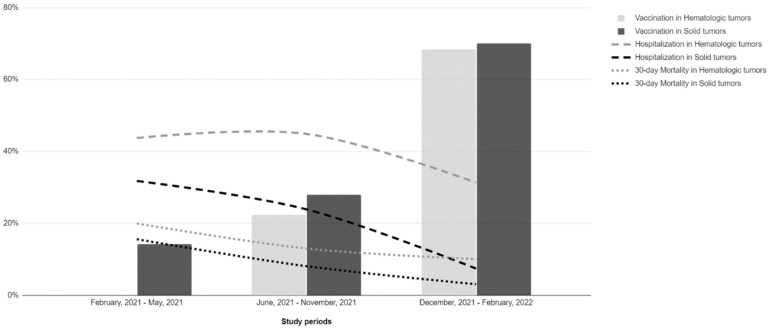
Vaccination status and outcomes in patients with hematologic malignancies and solid tumors according to study periods.

Patients who were infected in the first and second wave had either not been vaccinated or had lower rates of vaccination and higher rates of mortality and hospitalization compared to those infected in the Omicron wave (see Figure 1). In patients with solid tumors, the rate of hospitalization was significantly lower during Omicron (7.5%) compared to the other two waves (32% during the first wave and 24% during the second wave, *p* < 0.001). Mortality at 30 days was reduced from 16% and 8% during the first two waves, respectively, to 3% during Omicron (*p* < 0.001). Mechanical ventilation was reduced from 12% in the first wave to <1% in Omicron (*p* < 0.001). In hematologic patients, there was a significant reduction in the proportion of patients on mechanical ventilation from the first (38%) and second (29%) wave to the Omicron period (11%) (*p* < 0.005) (Table 2).

### 3.3. Main Outcomes

#### 3.3.1. Hospitalization

Overall, 152 patients (22%) were admitted to the hospital at COVID-19 diagnosis: 115 patients due to COVID-19 disease and 37 for other reasons. Another 28 patients had to be admitted later due to increased oxygen requirements. The proportion of hospitalization for any cause was higher in HM patients compared to ST patients (41% vs. 16%, respectively, *p* < 0.01), and the same is true for the days of hospital stay (13 vs. 9 days, *p* = 0.01).

#### 3.3.2. Invasive Mechanical Ventilation

A total of 186 patients (27%) needed low-flow oxygen therapy, and 32 (5%) required non-invasive ventilation. Additionally, 50 patients (7%) required invasive mechanical ventilation, and 54 (8%) were admitted to the COVID-19 Intensive Care Unit.

#### 3.3.3. Thirty-Day Mortality

Twenty-nine patients (4.2%) were lost to follow-up. These patients were excluded from the mortality analysis. The 30-day mortality was 8% (N = 51): 19 (37%) patients died due to bacterial complications (n = 11), invasive fungal infections (n = 6), or acute respiratory distress syndrome (n = 2). In four patients, death was related to the oncologic disease. Fifteen patients (31%) refused intubation, eleven (22%) were under terminal sedation, and two (4%) patients were transferred to another hospital.

Patients with an HM had a higher risk of being admitted to the ICU, were more likely to use invasive mechanical ventilation, and had a higher risk of death (Table S2).

Table 3 describes the crude ORs for the association of vaccination status with the different outcomes, stratified by type of neoplasia. Vaccinated patients with STs and COVID-19 had 70% less risk of being hospitalized and 60% less risk of progressing to severe or critical disease compared to unvaccinated patients with STs (*p* < 0.01). In the HM group, having 2 or 3 vaccine doses was associated with half the risk of hospitalization for any cause compared to unvaccinated patients (*p* = 0.04).

There were no significant differences in outcomes between the different types of vaccines used (AZ, Pfizer, Sputnik, and Sinovac). All patients with two or more doses of a vaccine were included in this analysis. The mRNA1273 and J&J vaccines were excluded due to the small number of individuals who had received them.

In patients with solid tumors, age, time of infection, recent chemotherapy, being vaccinated with at least 2 doses, and having any comorbidity, were all associated with higher odds of hospitalization. Having COVID-19 in the last wave was associated with lower odds of hospitalization, mortality, or invasive ventilation. In those with hematologic malignancies, there was no independent variable associated with mortality or hospitalization. However, infection during the first period was associated with higher odds of invasive ventilation (Table 4).

## 4. Discussion

Herein, we describe the clinical characteristics and outcomes of cancer patients with laboratory-confirmed COVID-19 after the rollout of the vaccines against SARS-CoV-2 in Mexico with the outcomes of a wider array of vaccines, including vector-based, mRNA, and whole inactivated virus vaccines.

The patients of this study were older than the median age of the general population (median age 52 yo), having a high proportion of comorbidities (44%), and 27% of the patients in this study had not received a single vaccine dose. Vaccinated patients were significantly older and had more comorbidities compared to unvaccinated patients. This is partly due to Mexico’s vaccination policies. Although patients with cancer were prioritized for vaccination in many countries [20], Mexico did not include immunosuppression as a priority criterion in the rollout of COVID-19 vaccines.

Vaccination reduced hospitalization in patients with STs, having a lesser impact in patients with HMs. SARS-CoV-2 immunity induced by vaccines has changed the natural history of COVID-19 by reducing the progression and mortality of the disease. However, cancer patients, despite a full vaccination scheme, remain at risk of breakthrough infections and adverse outcomes associated with COVID-19 [21]. In the National COVID Cohort Collaborative (N3C) study, 6860 breakthrough cases were identified, among which 1460 (21.3%) were patients with cancer. ST and HM patients had significantly higher risks for breakthrough infection (odds ratios [ORs] 1.12, 95% CI, 1.01 to 1.23 and 4.64, 95% CI, 3.98 to 5.38) and severe outcomes (OR 1.33, 95% CI, 1.09 to 1.62 and 1.45, 95% CI, 1.08 to 1.95) compared with non-cancer patients. Breakthrough infection risk was reduced after the second dose of the vaccine for all cancers (OR = 0.04; 95% CI = 0.04 to 0.05) [11].

Several reports have shown that both vaccinated and unvaccinated patients with HMs have higher rates of intensive care admission, use of mechanical ventilation, and hospitalization compared to those with STs (OR = 2.00, 95% CI = 1.41–2.83 and OR = 2.42, 95% CI = 1.82–3.22, respectively) [22]. The worse outcomes in patients with HMs are due to these individuals having poor vaccine-induced immunity and more severe immunosuppression. Herishanu et al. published results regarding serologic responses to the BNT162b2 COVID-19 vaccine in a cohort of chronic lymphocytic leukemia (CLL) patients, showing that responses in these patients were poor. Poor serologic response to vaccines was associated with older age, diagnosis of CLL, recent treatment with anti-CD20 antibodies, and less than 60 days between the end of treatment and COVID-19 vaccination [23]. Although we could not measure neutralizing antibodies to establish the serologic response to the vaccines, we did find more severe outcomes in the HM group compared to the ST group, which supports the notion of more profound immunosuppression in the HM group. However, despite these patients having a poorer serologic response, Jimenez et al. reported preserved cellular response in patients with a blunted humoral response (mostly patients on anti-CD20 treatment) vaccinated with mRNA vaccines, highlighting a benefit from vaccination in hematologic patients [24]. Moreover, Bahremand et al. analyzed the effect of mRNA booster vaccines in severely immunosuppressed patients in a population-based study, and although the hospitalization rate of the immunosuppressed patients was higher than that of the non-immunosuppressed patients, they found additional benefits for those with severe illness from a third mRNA dose in the most extremely vulnerable individuals [25].

In our study, the overall mortality was 12.1%, and the 30-day mortality was 8%. In the pre-vaccination era, at our institution, the reported 30-day mortality was 24% [26]. Prior to SARS-CoV-2 vaccination, mortality rate was exceptionally high in patients with active cancer and COVID-19. During the first wave, mortality rates were commonly reported to be around 40%, decreasing to approximately 25% in the following waves in European countries in 2021 [20,27]. After the introduction of the vaccine and new antiviral treatment strategies, mortality in patients with cancer improved, depending on the type of targeted treatment received, cancer staging, and oncologic treatment, amongst other factors [28]. Other cohorts have showed mortality rates of 10–12% in oncologic patients after the rollout of vaccines [29].

We describe a reduction in all outcomes for patients with solid tumors infected during the third period, related to the Omicron variant. This corresponds to a variant with a reduced virulence and disease severity [30], but it is also a period associated with improved access to vaccines, and antiviral treatment. In our sample, the Omicron period was associated with a much higher proportion of fully vaccinated patients (70% compared to 11% and 27% in the first and second period, respectively), and 93% of patients who received remdesivir were administered this medication during Omicron. Although we used multiple regression to evaluate variables associated with the outcomes, it is difficult to separate the individual effect of each of those variables in the improvement of outcomes.

The reduction in unfavorable outcomes was not so marked in the patients with hematologic malignancies, even during Omicron. Regarding clinical outcomes, some studies have reported some improvement in hospitalization and mortality in vaccinated patients with hematologic malignancies. However, as previously mentioned, these studies evaluated mostly mRNA vaccines [11,13]. The lack of improvement in our vaccinated hematologic patients could be due to the lower efficacy of other non-mRNA vaccines. It is also probably associated with other factors, such as more profound immunosuppressive status, chronic wasting, and malnutrition. We report results mostly concerning patients with advanced-stage disease, differing from other cohorts reported, and disease stage could also affect the response to vaccines [26,31].

According to WHO data released on March 03, 2023, 180 vaccines have been approved for clinical trials, and 199 vaccines are under preclinical trial [32]. The initial data on vaccine efficacy in cancer patients were obtained from small prospective observational studies focused on immunological or antibody responses [33]. In a meta-analysis of COVID-19 vaccines in immunocompromised patients, it was found that 94% of studies used mRNA vaccines, 20% used viral vectors, and 5% used whole inactivated virus vaccines [34]. Among the most widely used WHO-approved vaccines are BNT162b2 (an mRNA vaccine from Pfizer–BioNTech), mRNA-1273 (an mRNA vaccine from Moderna–NIAID), AZD1222 (a viral vector vaccine from AstraZeneca–University of Oxford), and BIBP-CorV (an inactivated vaccine from Beijing Institute of Biological Products), with these vaccines reportedly having efficacy values of 95%, 94%, 70%, and 79%, respectively [35]. In a study comparing the immune response of different vaccine platforms (mRNA versus non-mRNA) in cancer patients, those who received the mRNA-1273 vaccine had the highest median levels of S-RBD IgG and Nab, followed by those who received BNT162b, AZS1222, and BBIBP-CorV/Coronavac [36]. We did not find any differences in the outcomes by vaccine type. This could be due to the low proportion of patients vaccinated with mRNA vaccines (less than 25%). MRNA vaccines are, as discussed above, the most well-documented vaccines with respect to information on efficacy in immunosuppressed patients. In our cohort, almost 35% of individuals received vaccines for which there is very little information on efficacy in cancer patients (Sputnik—18%; AD5-nCOV—6%; and Sinovac—10%). Regarding the Sinovac vaccine, Simsek et al., in their retrospective study, suggest adequate vaccine efficacy in patients with solid tumors vaccinated with two to three doses of the Sinovac vaccine [37]; however, other studies have reported poorer response, with enhanced immunogenicity after a third mRNA dose in patients who have either received the Sinovac vaccine or heterologous Sinovac/AZD1222 primary regimens [38,39]. Almost no reports exist on Sputnik-V and AD5-nCOV efficacy in patients with cancer. Our results would seem to support prioritizing mRNA vaccination in patients with cancer. However, we were not able to collect information on the type of vaccine used for almost 40% of the vaccinated patients, which might have affected the analysis. More studies are needed to identify the impact of vaccines other than mRNA vaccines in cancer patients, especially in low- and middle-income countries.

In this study, 27% of the individuals did not receive a single vaccine dose at the time of COVID-19 diagnosis. This might be because they did not have access to the vaccine, but it also could be due to vaccine refusal or lack of recommendation by their oncologists. Although we did not measure vaccination acceptance in our population, there are reports describing low vaccination coverage and vaccination acceptance in oncologic patients [40]. In a prior study, COVID-19 vaccination acceptance in the general population in Mexico was 62.3%, refusal was 28.2%, and hesitancy was 9.5%. Refusal and hesitancy were associated with being female, older age, lower educational and socioeconomic status, and working in the informal sector [41].

This study has some limitations. Due to its retrospective nature, there could be some unmeasured confounders not included in the analysis. Also, the data reported only represent one cancer center. However, INCan is a reference hospital receiving patients from all over the country, and the data presented here represent the largest sample published in Latin America. This study also provides information on different types of COVID-19 vaccines, rather than only focusing on mRNA vaccines, for patients with cancer, including real-world data. Another limitation of our study is that we could not measure serologic response to vaccination, but we are able to describe the clinical outcomes of the disease in vaccinated and non-vaccinated individuals, which usually are the main outcomes in vaccine trials. Also, we did not collect information on prior COVID-19 infection, which could affect the immune response of individuals. However, this is probably random bias, and the proportion of individuals with prior infection might be similar in both groups of fully vaccinated and not fully vaccinated patients. Finally, we report a population where less than 50% were fully vaccinated subjects, but this reflects the status of the majority of patients with cancer in Mexico, where the rollout of the vaccines did not prioritize patients with cancer, and nor were they prioritized to receive a specific type of vaccine.

In summary, we have reported real-world data on SARS-CoV-2 infections in patients with cancer pertaining to after the COVID-19 vaccines arrived in Mexico early in 2021. As in other reports, vaccines decreased the severity of COVID-19 disease, especially in patients with STs compared to those with HMs. The Omicron variant was also associated with decreased severity, and recent chemotherapy was associated with worse outcomes in patients with both hematologic and solid tumors. Although effectiveness is reduced in immunosuppressed patients, vaccination is still an essential strategy, and access to vaccination campaigns in patients with cancer needs to be prioritized as part of other interventions, such as antiviral treatment and interdisciplinary management, to ensure optimal infection control and adequate timing of the oncologic treatment.

## Figures and Tables

**Table 1:** Baseline characteristics of patients by type of neoplasia.

	All Patients N = 691 (%)	Solid Tumor N = 524, (%)	Hematologic Malignancy N = 167, (%)	*p* Value
Female sex	442 (64)	373 (71)	69 (41)	<0.01
Median age (IQR) years	52 (42–63)	52 (43–63)	49 (35–62)	0.01
At least one comorbidity	305 (44)	242 (46)	63 (38)	0.05
Diabetes	105 (19.7)	81 (16)	24 (14)	0.7
High blood pressure	142 (26.6)	116 (22)	26 (16)	0.6
Obesity	143 (26.8)	120 (23)	23 (14)	0.01
Patients living with HIV	25 (4.7)	11 (2)	14 (8)	<0.001
Chronic renal failure	7 (1.3)	2 (0.3)	5 (3)	0.003
Dyslipidemias	23 (4.3)	13 (2.4)	10 (6)	0.02
Lung disease	33 (6.2)	27 (5)	6 (4)	0.4
Others ^a^	52 (9.7)	36 (7)	16(10)	0.2
Active cancer	583 (84)	451 (86)	132 (79)	0.02
Cancer-free	113 (16)	78 (15)	35 (20)	0.06
Palliative care	54 (8)	51 (10)	3 (2)	<0.01
Active chemotherapy	449 (65)	327 (62)	122 (73)	0.01
Chemotherapy in the last month	265 (38)	182 (35)	83 (50)	<0.001
Immunotherapy	45 (7)	37 (7)	8 (5)	0.3

^a^ neurologic, gastrointestinal, rheumatologic, endocrine, and psychiatric comorbidities.

**Table 2:** Odds ratios for mortality and hospitalization comparing Omicron to the first and second wave.

Odds Ratios for Mortality and Hospitalization Comparing Omicron to the First and Second Wave				
	OR (95%CI)			
	Hospitalization		30-Day Mortality	
Study Periods	Hematologic Tumors	Solid Tumors	Hematologic Tumors	Solid Tumors
3rd vs. 2nd wave	0.56 (0.28–1.14)	0.26 (0.14–0.46)	0.75 (0.25–2.21)	0.36 (0.15–0.88)
3rd vs. 1st wave	0.59 (0.19–1.73)	0.17 (0.08–0.35)	0.45 (0.11–1.89)	0.17 (0.07–0.46)

**Table 3:** Crude outcomes by vaccination status and type of neoplasia (solid and hematologic neoplasia).

	Fully Vaccinated (2–3 Doses) (n, %)	Unvaccinated (0–1 Doses) (n, %)	OR (95%CI)	*p* Value
Patients with Solid Tumors (N = 466)				
	20 (7.9)	50 (23.2)	0.2 (0.1–0.4)	<0.01
Invasive mechanical ventilation	5 (1.9)	7 (3.2)	0.7 (0.1–2.3)	0.5
30-day mortality	9 (3.6)	17 (8.0)	0.4 (0.1–1.00)	0.06
Patients with hematologic disease (N = 155)				
Hospitalization at COVID-19 diagnosis	27 (33.75)	35 (46.6)	0.5 (0.2–0.9)	0.04
Invasive mechanical ventilation	9 (11.2)	18 (24.0)	0.4 (0.1–1.0.)	0.05
30-day mortality	7 (9.2)	8 (10.9)	0.8 (0.2–2.4)	0.7

**Table 4:** Multivariate analysis for hospitalization, invasive mechanical ventilation, and 30-day mortality in patients with solid cancer and hematologic neoplasias.

Solid Cancer Patients												
Outcome	Hospital Admission				Invasive Mechanical Ventilation				30-Day Mortality			
	Yes n = 82 (%)	No n = 381 (%)	aOR (95%CI)	*p* Value	Yes n = 19 (%)	No n = 444(%)	aOR (95%CI)	*p* Value	Yes n = 32 (%)	No n = 417 (%)	aOR (95%CI)	*p* Value
Age in years	59 (49–68)	52 (42–61)	1.03 (1.009–1.05)	0.005	57 (54–71)	52 (43–63)	1.01 (0.97–1.07)	0.45	64 (54–72)	52 (43–62)	1.04 (1.00–1.07)	0.016
Chemotherapy in the last month	41 (50)	140 (37)	2.78 (1.53–5.05)	0.001	9 (47)	172 (39)	3.21 (0.90–11.4)	0.071	16 (50)	162 (39)	2.25 (0.96–5.29)	0.06
Fully vaccinated (2–3 doses)	23 (34)	196 (56)	0.41 (0.21–0.80)	0.010	5 (42)	214 (53)	0.7 (0.1–2.9)	0.63	9 (35)	202 (53)	0.47 (0.18–1.26)	0.139
Waves: 1	21 (26)	37 (10)	0.28 (0.16–0.47)	<0.001	8 (42)	50 (11)	0.24 (0.08–0.66)	0.006	10 (31)	46 (11)	0.37 (0.18–0.74)	0.005
2	39 (48)	102 (27)			9 (47)	132 (30)			13 (41)	123 (30)		
3	22 (27)	242 (64)			2 (11)	262 (59)			9 (28)	248 (59)		
At least one comorbidity	50 (61)	172 (45)	2.20 (1.16–4.17)	0.015	12 (63)	210 (47)	3.3 (0.76–14.69)	0.08	17 (53)	198 (48)	1.2 (0.48–2.94)	0.689
Hematologic patients												
Age in years	53 (36–63)	47 (33–60)	1.01 (0.99–1.03)	0.26	52 (45–66)	48 (33–60)	1.02 (0.99–1.05)	0.08	48 (33–60)	53 (46–64)	1.02 (0.98–1.05)	0.25
Chemotherapy in the last month	33 (55)	49 (48)	1.4 (0.71–2.82)	0.31	20 (65)	62 (47)	2.5 (1.005–6.64)	0.049	12 (63)	65 (49)	3.03 (0.90–10.24)	0.073
Fully vaccinated (2–3 doses)	22 (40)	55 (57)	0.58 (0.26–1.27)	0.17	9 (33)	68 (55)	0.62 (0.21–1.78)	0.37	7 (47)	66 (52)	0.65 (0.18–2.03)	0.511
Waves: 1	7 (11)	9 (9)	0.62 (0.31–1.23)	0.17	6 (19)	10 (8)	0.35 (0.15–0.84)	0.019	3 (16)	12 (9)	1.09 (0.36–3.25)	0.85
2	22 (37)	26 (25)			14 (45)	34 (26)			6 (32)	38 (28)		
3	31 (52)	68 (66)			11 (35)	88 (67)			10 (53)	84 (63)		
At least one comorbidity	23 (38)	38 (37)	0.95 (0.44–2.03)	0.90	11 (35)	50 (38)	0.55 (0.20–1.54)	0.26	8 (42)	48 (36)	1.54 (0.48–4.92)	0.46

## Data Availability

Data are available as an excel file in the Supplementary Material.

## Supplementary Materials

The following supporting information can be downloaded at: https://www.mdpi.com/article/10.3390/vaccines12101163/s1, Figure S1: Distribution of cancer types in patients with solid tumors (left) and hematologic malignancies (right); Figure S2: COVID-19 severity by type of neoplasia and vaccination status; Table S1: Characteristics of patients according to their vaccination status; Table S2. COVID-19-related outcomes by type of neoplasia; Supplementary Materials: Data.
