# Supplementary material for: SARS-CoV-2 Vaccination in Patients with Cancer and COVID-19 in Mexico
Source: Vaccines (Basel). 2024 Oct 12;12(10):1163. doi: 10.3390/vaccines12101163 (PMC11512203; doi:10.3390/vaccines12101163)
Supplement: Supplementary file 1 [file vaccines-12-01163-s001.zip › vaccines-3194953 - Supplementary Materials Final.pdf]

Supplement material:

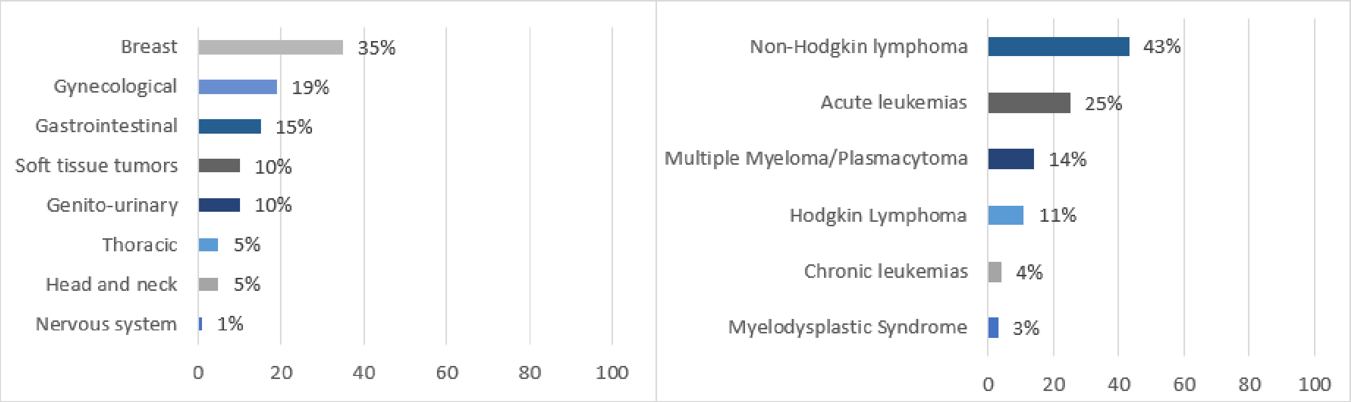

Figure S1. Distribution of cancer type in patients with solid tumors (left) and hematologic malignancies (right).

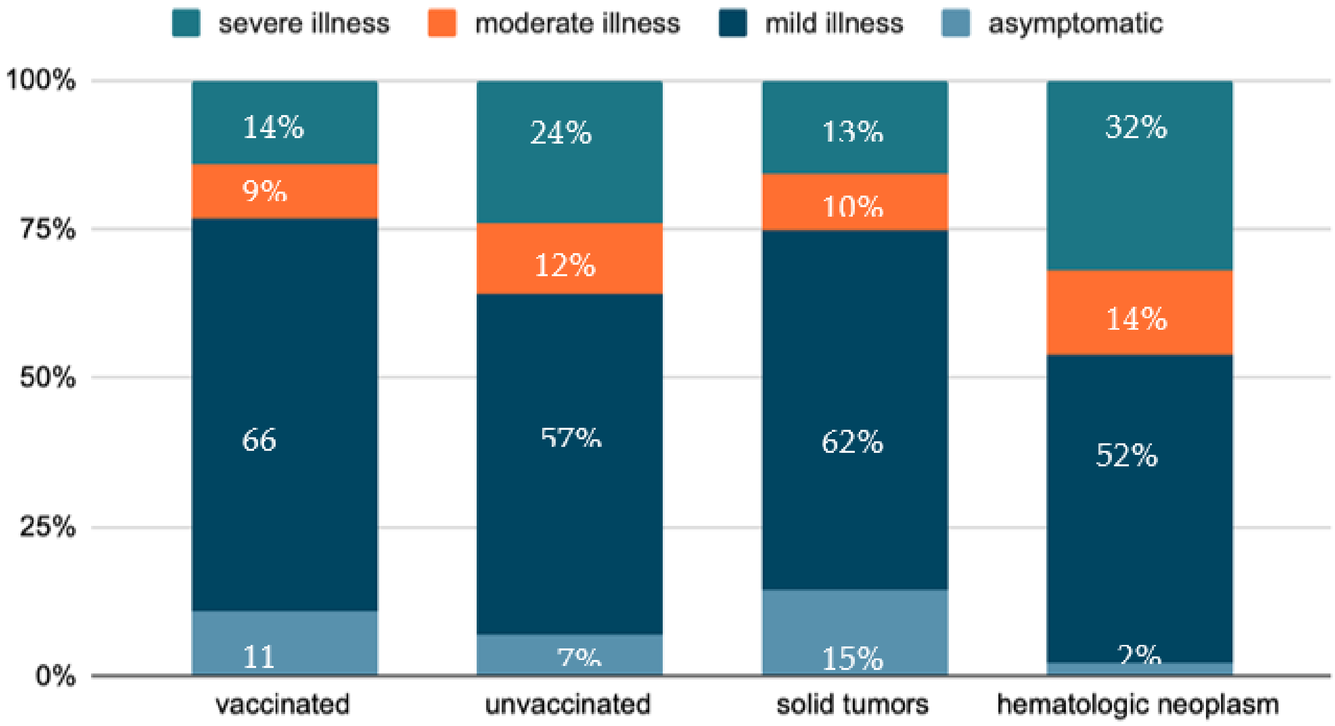

Figure S2. COVID-19 severity by type of neoplasia and vaccination status.

Table S1. Characteristics of patients according to their vaccination status.

	**Unvaccinated (0-1 doses) n= 290**	**Fully Vaccinated (2- 3 doses) n=331**	**P value**
Median age (IQR) years	47 (37-58)	55 (47-66)	<0.01
Female	99 (34)	113 (34)	1.0
Diabetes	32 (11)	60 (18)	0.012
High blood pressure	41 (14)	91 (28)	<0.001
Patients living with HIV	9 (3)	13 (4)	0.579
Solid Tumors	215 (74)	251 (76)	0.6
Hematologic malignancy (HM)	75 (26)	80 (24)	0.6
Cytotoxic chemotherapy	177 (61)	162 (49)	<0.001
Rituximab	21 (7)	33 (10)	0.2

Table S2. COVID-19 related outcomes by type of neoplasia.

	**Solid tumors (N=524)**	**Hematologic malignancy (N=167)**	**OR (95%CI)**	**P value**
Hospitalization at diagnosis due to COVID-19 (N=115)	82 (15.6%)	61 (36.5%)	3.1 (IC 2.1 – 4.5)	<0.001
Hospitalization due to COVID-19 progression (N=28)	15 (2.8%)	13 (7.7%)	2.8 (IC 1.3 -6.1)	<0.001
Invasive Mechanical ventilation (N=50)	19 (4%)	31 (19%)	6.8 (IC 3.5- 13.22)	<0.001
Admission to the Intensive Care Unit (N=54)	23 (4%)	31 (19%)	5.9 (IC 2.9-9.2)	<0.001
Death (N=51)[a]	32 (6.2%)	19 (11.8%)	2.0 (IC 1.1 – 3.6)	<0.001

a: mortality at 30-days.
